# Regulation of Anthocyanin Biosynthesis in Purple Leaves of Zijuan Tea (*Camellia sinensis* var. *kitamura*)

**DOI:** 10.3390/ijms18040833

**Published:** 2017-04-19

**Authors:** Lingxia Wang, Dezhuo Pan, Meng Liang, Yakubu Saddeeq Abubakar, Jian Li, Jinke Lin, Shipin Chen, Wei Chen

**Affiliations:** 1 College of Life Sciences, Fujian Agriculture and Forestry University, Fuzhou 350002, China; wang_lx0218@163.com (L.W.); pdz_006@163.com (D.P.); liangyiyu2012@163.com (M.L.); ay.saddeeq@yahoo.com (Y.S.A.); li123456jian@126.com (J.L.); 2 College of Life Sciences, Ningxia University, Yinchuan 750021, China; 3 College of Forestry, Fujian Agriculture and Forestry University, Fuzhou 350002, China; 4 Anxi College of Tea Sciences, Fujian Agriculture and Forestry University, Fuzhou 350002, China; ljk213@163.com

**Keywords:** anthocyanin accumulation, differential proteins, isobaric tag for relative and absolute quantification (iTRAQ), Zijuan tea

## Abstract

Plant anthocyanin biosynthesis is well understood, but the regulatory mechanism in purple foliage tea remains unclear. Using isobaric tag for relative and absolute quantification (iTRAQ), 815 differential proteins were identified in the leaves of Zijuan tea, among which 20 were associated with the regulation of anthocyanin metabolism. We found that the abundances of anthocyanin synthesis-related enzymes such as chalcone synthase, chalcone isomerase, dihydroflavonol 4-reductase and anthocyanin synthetase, as well as anthocyanin accumulation-related UDP-glucosyl transferase and ATP-binding cassette (ABC) transporters in the purple leaves were all significantly higher than those in the green leaves. The abundances of the transcription factors bHLH and HY5, regulating anthocyanin biosynthesis at transcriptional level were also obviously higher in purple leaves than those in green leaves. In addition, bifunctional 3-dehydroquinate dehydratase and chorismate mutase in purple leaves were distinctly higher in abundance compared to green leaves, which provided sufficient phenylalanine substrate for anthocyanin synthesis. Furthermore, lignin synthesis was found to be reduced due to the lower abundances of cinnamoyl-CoA reductase 1, peroxidase 15 and laccase-6, which resulted in increase of intermediates flow into anthocyanin synthesis pathway. The physiological data were consistent with proteomic results. These four aspects of biosynthetic regulation contribute to anthocyanin accumulation in purple leaves of Zijuan tea.

## 1. Introduction

Anthocyanins are natural water-soluble pigments belonging to the family of flavonoids. Thus far, more than 250 anthocyanins have been identified in plants [1]. Anthocyanins can efficiently remove free radicals and induce a strong antioxidant activity in cells from various organisms due to containing numerous phenolic hydroxyl groups [2]. Therefore, anthocyanins have been considered as functional substances in anti-aging, suppressing cancer tumors, reducing blood lipid levels, protecting the liver, and performing other physiological effects in humans [3,4,5,6].

Plant anthocyanins accumulate in many organs, such as roots, leaves, flowers, and fruits. They are stored in cell vacuoles and display different colors in form of cyanine glycosides after glycosylation, methylation, and acetylation from an anthocyanin monomer [7,8]. For example, the purple foliage tea, a special and scarce tea tree germplasm resource, appears red, purple, or reddish violet in color due to the accumulation of anthocyanins. The concentration of anthocyanin in purple leaves reaches 707 μg·g^−1^ Dry Weight (DW) in Zijuan tea (*Camellia sinensis* var. *kitamura*), which is 10-fold higher than that of an ordinary tea [9,10].

The genetic characteristics and biochemical properties involved in anthocyanin biosynthesis have been widely explored using model plants, including *Arabidopsis thaliana* [11], *Petunia hybrid* [12], and *Zea mays* [13]. The anthocyanin concentration in purple foliage tea has been investigated. Jiang et al. [9] identified four anthocyanins in purple leaves of Zijuan tea. Kerio et al. [14] analyzed the components of anthocyanins in purple leaves of ‘Kenyan’ tea and found that the anthocyanin concentration in purple leaves was evidently higher than that in green leaves. Five kinds of important anthocyanins, especially scabiolide, are also present in the tea variety. Saito et al. [15] isolated six components of anthocyanins from “Sunrouge” tea (*Camellia taliensis* × *Camellia sinensis*) exhibiting various biological activities, which were identified through liquid chromatography and nuclear magnetic resonance spectroscopy. Furthermore, numerous genes involved in the anthocyanin metabolic pathway have been cloned, and the relationship between the expression patterns of these genes and the accumulated anthocyanins has been reported in the purple leaves of Zijuan tea and Zhejiang safflower camellia plants [16,17]. Nine genes related to anthocyanin metabolism in Zhejiang safflower camellia, including phenylalanine ammonia lyase (*PAL*), chalcone synthase (*CHS*), flavanone 3-hydroxylase (*F3H*), and dihydroflavonol reductase (*DFR*), have been identified by transcriptome sequencing [17]. Park et al. [18] examined the gene expression sequence tags of *CHS*, *F3H*, and leucoanthocyanidin reductase in young leaves and found that their expression levels were significantly higher than those in old leaves of Zijuan tea. Yang et al. [19] cloned the transcription factor gene *CsMYB1* in Zijuan tea leaves and analyzed this gene with quantitative real-time PCR (qRT-PCR). They confirmed that *CsMYB1* is essential for anthocyanin accumulation and plays an important role in flavonoid metabolism.

For purple foliage tea trees, juvenile leaves are purple, and mature leaves gradually turn green as growth continues. However, the regulatory mechanism of anthocyanin metabolism in the purple leaves of the tea trees also remains unclear. In the present study, anthocyanin-rich Zijuan tea was selected as a material to explore the regulatory mechanism of anthocyanin metabolism in the purple leaves. Isobaric tag for relative and absolute quantification (iTRAQ) was conducted to obtain the profile of protein expression changes between purple and green leaves of Zijuan tea. This study aimed to explain anthocyanin biosynthesis and its regulatory network in Zijuan tea.

## 2. Results

### 2.1. Changes in Protein Abundance between Purple and Green Leaves

In this study, 2352 proteins were identified by iTRAQ, among which 2252 were non-redundant. Approximately 815 proteins were quantified in purple leaves, in which 544 showed more than 1.5-fold changes in abundance and 271 exhibited less than 0.6-fold changes in abundance as compared to the proteins in green leaves. Of these 544 proteins, 20 were involved in anthocyanin metabolism and regulation (Table 1). A total of 10 proteins (spots 1–10) that were directly involved in anthocyanin synthesis, modification, and transshipment were upregulated. In addition, the other 10 proteins (spots 11–20), indirectly involved in anthocyanin regulation, were also upregulated except spots 17, 19 and 20.

### 2.2. Changes in Chlorophyll, Proanthocyanidin, Anthocyanin and Lignin Concentrations in Leaves

**Figure 1:**
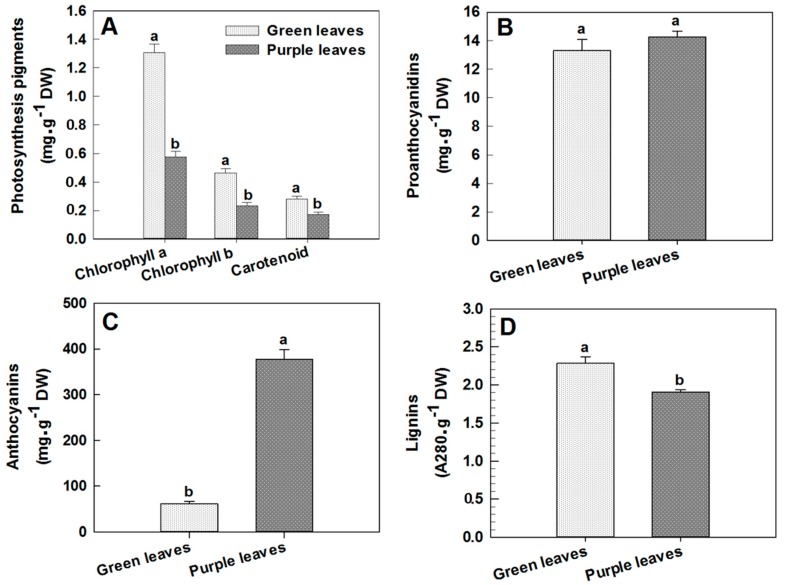
The concentrations of photosynthetic pigments, proanthocyanidins, anthocyanins and lignins in green and purple leaves of Zijuan tea. (**A**) the content of photosynthetic pigments; (**B**) the content of proanthocyanidins; (**C**) the content of anthocyanin; (**D**) the content of lignin. Error bars indicate standard errors of three biological replicates. Different lowercase letters above the bars indicate a significant difference between green and purple leaves samples at *p* < 0.05.

The purple leaves of Zijuan tea gradually become green as the plant attains its different developmental stages. This happens due to changes in the concentration of different pigments in the leaf cells with age advancement. In the present study, we found that the concentrations of chlorophyll a, chlorophyll b and carotenoid in purple leaves of this plant were 0.57, 0.23 and 0.17 mg·g^−1^ DW respectively, which were significantly decreased by 56.46%, 50.00% and 39.29% compared to those of green leaves, respectively (*p* < 0.05, Figure 1A). However, the proanthocyanidin concentration in purple leaves was 14.27 mg·g^−1^ DW, which was 7.05% higher than that in green leaves, although the analysis of variance (ANOVA) indicated that this difference was not significant (Figure 1B). The total anthocyanin concentration in purple leaves of Zijuan tea was found to be 377.13 μg·g^−1^ DW, which was 6.2-fold that of green leaves (60.38 μg·g^−1^ DW; Figure 1C). Meanwhile, the lignin concentration in purple leaves was 1.91 A_280_·g^−1^, which was 16.59% lower than that in green leaves, and ANOVA results showed that this difference was significant (*p* < 0.05, Figure 1D).

### 2.3. Changes in PAL, Chalcone Isomerase (CHI) and UDP-Glycosyl Transferase (UGT) Activities between Purple and Green Leaves

**Figure 2:**
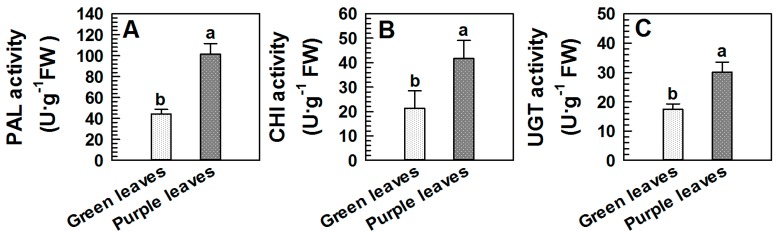
The activities of PAL, CHI and UGT in the green and purple leaves of Zijuan tea. (**A**) The activity of PAL; (**B**) the activity of CHI; (**C**) the activity of UGT. FW: fresh weight; PAL: phenylalanine ammonialyase; CHI: chalcone isomerase; UGT: UDP-glycosyl transferase. Error bars indicate standard errors of three biological replicates. Different lowercase letters above the bars indicate a significant difference between green and purple leaves samples at *p* < 0.05.

PAL is the first enzyme in the biosynthesis of anthocyanin metabolism. The enzyme activity was 2-fold higher in purple leaves than in green ones (Figure 2A). In addition, CHI is another important regulatory enzyme in this biosynthesis pathway. We found that CHI activity in the purple leaves was significantly increased compared to the green ones (*p* < 0.05, Figure 2B). Furthermore, UGT, the final enzyme in anthocyanin biosynthesis, catalyzes glucosyl transfer from UDP-glucose to 3-hydroxyl group to form stable cyanine glucosides. It was almost 2-fold greater in the purple leaves than in the green ones, and the ANOVA result indicated that this difference was significant (*p* < 0.05, Figure 2C).

### 2.4. qRT-PCR Analyses for Differentially Expressed Proteins Related to Anthocyanin Metabolism in Purple Leaves

**Figure 3:**
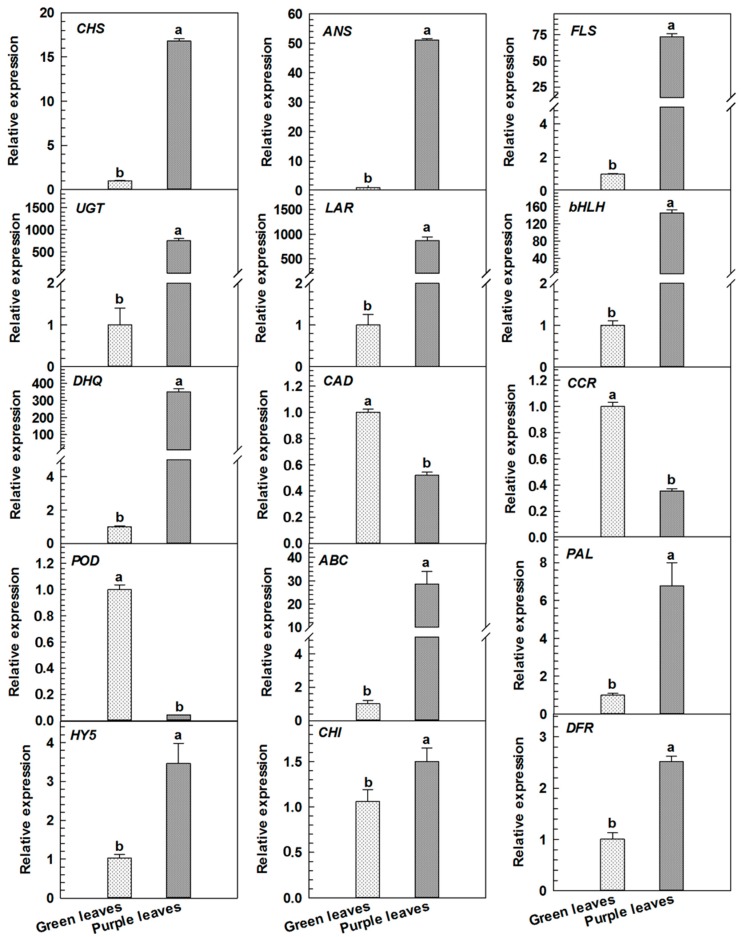
Real-time PCR analyses of genes of differentially expressed proteins in green and purple leaves of Zijuan tea. *CHS*: chalcone synthase; *CHI*: chalcone isomerase; *DFR*: dihydroflavonol 4-reductase; *ANS*: anthocyanindin synthase; *FLS*: flavonol synthase; *UGT*: UDP-glycosyl transferase; *LAR*: leucoanthocyanidin reductase; *DHQ*: 3-dehydroquinate dehydratase; *CAD*: cinnamyl alcohol dehydrogenase; *CCR*: cinnamoyl-CoA reductase 1; *POD*: peroxidase; *ABC*: ATP-binding cassette; *PAL*: phenylalanine ammonia lyase. Error bars indicate standard errors of three biological replicates. Different lowercase letters above the bars indicate a significant difference between green and purple leaves samples at *p* < 0.05.

In the present study, the transcriptional levels of 10 differential proteins associated with the regulation of anthocyanin metabolism in purple leaves were examined. Table 1 showed these proteins as CHS (spot 1), CHI (spot 3), DFR (spot 4), anthocyanin synthase (ANS, spot 5), UDP-glycosyl transferase (UGT, spot 6), ATP-binding cassette (abc) transporter b family member 8 (ABC transporter B8, spot 7), flavonol synthase (FLS, spot 8), leucoanthocyanidin reductase (LAR, spot 9), bHLH (spot 11), transcription factor hy5 (HY5, spot 13), bifunctional dehydrogenation quinic acid dehydratase (DHQ, spot 14), cinnamoyl-CoA reductase (CCR, spot 17), cinnamic alcohol dehydrogenase (CAD, spot 18), and catalase 15 (peroxidase, POD, spot 20). PAL abundance in the purple leaves was as 1.3-fold that of green leaves (data not shown). Figure 3 showed that in qRT-PCR analysis, the expression levels of these genes, namely, *CHS*, *CHI*, *DFR*, *ANS*, *UGT*, *FLS*, *LAR*, *bHLH*, *HY5*, *DHQ*, *ABC* and *PAL* in purple leaves were greater than those in green leaves (*p* < 0.05). However, the expression levels of three genes *CCR*, *CAD*, and *POD* in purple leaves were markedly lower than those in green leaves (*p* < 0.05).

### 2.5. Immunoblotting Analysis of CHS

**Figure 4:**
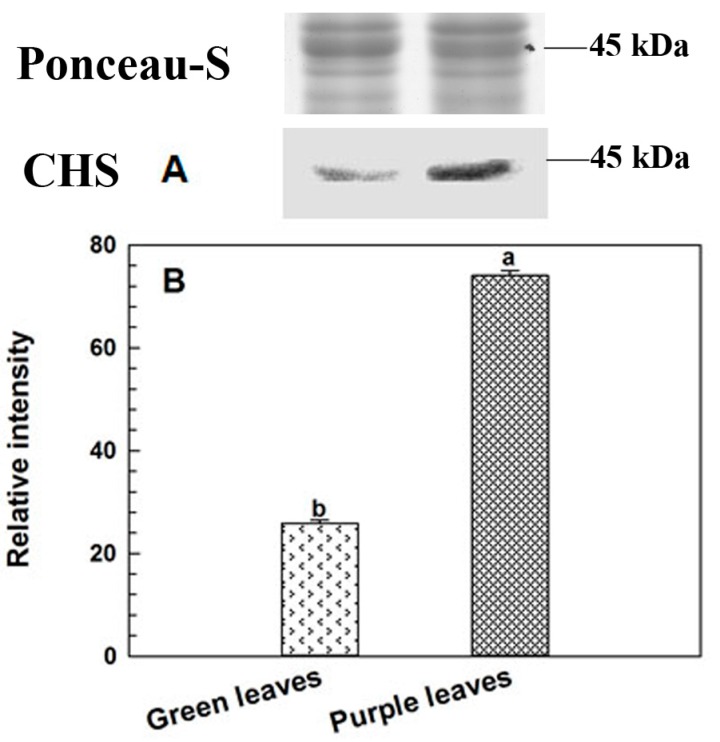
Western blot analysis of CHS in green and purple leaves of Zijuan tea. (**A**) the result of western blot; (**B**) relative intensity of CHS. The sample in each lane of western blot is a one-to-one correlation with the one in each bar of relative intensity. Ponceau-S staining was used as loading control. Error bars indicate standard errors of three biological replicates. Different lowercase letters above the bars indicate a significant difference between green and purple leaves samples at *p* < 0.05.

CHS is a key enzyme that regulates the conversion of 4-coumaric CoA to narigenin in anthocyanin biosynthesis pathway. In this study, the relative CHS abundance of iTRAQ analysis was confirmed by western blot. Ponceau-S staining was used as loading control to check consistent amount on each sample. The abundance level of CHS in green leaves was significantly lower than that in purple leaves (Figure 4). This result was consistent with iTRAQ quantitative data (Table 1).

## 3. Discussion

The anthocyanin pathway in higher plants is well understood, but the regulation of biosynthetic mechanism in anthocyanin-rich tea varieties remains unclear. Our findings in this study proposed that anthocyanin metabolism in Zijuan tea was regulated through four aspects as follows.

### 3.1. Regulation of Enzymes and Transporters in Anthocyanin Biosynthesis and Accumulation

A previous report indicated that both *PAL* expression and PAL activities were upregulated in purple leaves of the Zijuan tea tree [16]. In our proteomics data, PAL abundance in the purple leaves was slightly higher (1.3-fold) as compared to that in the green leaves, but the difference was insignificant. However, the enzyme activity was increased significantly in the purple leaves (Figure 2A). We suggested that the increased anthocyanin biosynthesis was mainly due to increased PAL activity, not the enzyme abundance.

In petunia, introduction of cDNA of an antisense *CHS* inhibits flower pigmentation that allows the successful change in its color [20]. Fukusaki et al. [21] found that the flower color of *Torenia hybrida* is modulated from blue to white and pale colors through RNA interference against *CHS*. Chalcone isomerase (CHI, spots 2 and 3) is another important regulatory enzyme in anthocyanin biosynthesis. Halbwirth et al. [22] reported that an increase in CHI activity resulted in anthocyanin accumulation in developing strawberries (*Fragaria* × *ananassa*). It is well known that flavonoids eventually transform into blue cyanidin, blue-purple delphinidin and red pelargonidin pigments, respectively. DFR is a key enzyme that regulates metabolic processes involved in anthocyanin or flavonol synthesis. Dihydroflavonol can be catalyzed by DFR and FLS to produce leucocyanidin and flavonol, respectively. Increment of DFR at both the protein abundance and mRNA expression level may induce the formation of colorless leucoanthocyanidins, which are converted by ANS to colored anthocyanidins. ANS is the last key enzyme in anthocyanin synthesis, directly affecting anthocyanin accumulation. Reddy et al. [23] reported that the overexpression of the *ANS* gene in rice can significantly increase anthocyanin concentration. In the present study, the abundances of CHS, CHI, DFR, and ANS were all higher in purple leaves compared to their green leaves in Zijuan tea, which was consistent with their transcriptional expression levels. We found that the anthocyanin concentration of purple leaves was also significantly higher than that of green leaves, suggesting that higher abundance and transcriptional levels of CHS, CHI, DFR, and ANS in purple leaves contributed to the increased anthocyanin synthesis.

UGT is the last enzyme in the anthocyanin biosynthetic pathway. Our result showed that both UGT abundance and activity in purple leaves were significantly higher than those in green leaves (Table 1, Figure 2C), and the mRNA relative expression of UGT in purple leaves was also higher compared to green leaves which was consistent with the previous report [16]. This observation was practically responsible for the stability and accumulation of anthocyanin in purple leaves. Upon their synthesis in cytosol and endoplasmic reticulum membrane system, anthocyanins are finally transported into the vacuoles via ABC transporters located in tonoplast [24,25]. The present study established that the abundance of the ABC transporter B8 in purple leaves was significantly higher than that in green leaves, which was consistent with its mRNA expression level (Table 1, Figure 3). This finding suggested that the protein might be actively involved in the transport of anthocyanin from the cytoplasm to vacuoles; as a consequence, anthocyanins were accumulated in cell vacuoles.

### 3.2. Regulation of Transcription Factors in Anthocyanin Biosynthesis

Anthocyanin synthesis can be directly regulated by key enzymes such as CHS, CHI, DFR, and ANS. However, their gene expression levels are regulated by transcription factors such as R2R3-MYB, bHLH, WD40, and HY5 [26]. For example, bHLH in the form of a dimer can firmly bind to DNA after R2R3-MYB is recognized by anthocyanin biosynthetic genes [27]; thus, the activity of gene promoters is enhanced [28]. In purple leaves of Zijuan tea, the MYB–bHLH–WDR complex regulates anthocyanin accumulation by activating mRNA expression of *F3H*, *DFR* and *ANS* [29]. In the present study, two proteins were identified as bHLH (Table 1). The abundance levels of bHLH 66-like and bHLH 135 in purple leaves were significantly higher than those in green leaves of Zijuan tea, and the mRNA expression level of bHLH was also higher in purple leaves. However, the abundance levels of R2R3-MYB and WD40 were not significantly different. These results implied that bHLH may be the major transcription factor for anthocyanin biosynthesis genes in purple leaves, promoting anthocyanin synthesis. Another protein, namely long hypocotyl5 (HY5), was also characterized as a transcription factor. Both the abundance and mRNA expression level of HY5 in purple leaves were also significantly higher than those in green leaves. HY5 acts as a downstream factor of multiple families of photoreceptors and promotes photomorphogenesis [30]. This protein can also regulate gene expression by directly binding to gene promoter regions in anthocyanin synthesis [31]. Shin et al. [32] reported that HY5 also regulates anthocyanin biosynthesis by inducing the transcriptional activation of the MYB75/PAP1 transcription factor in *Arabidopsis.* Therefore, the higher abundance of bHLH and HY5 might enhance the expression of anthocyanin biosynthetic genes. These results were confirmed by qRT-PCR analysis and anthocyanin concentration determination.

### 3.3. Regulation of Substrates in Anthocyanin Biosynthesis

**Figure 5:**
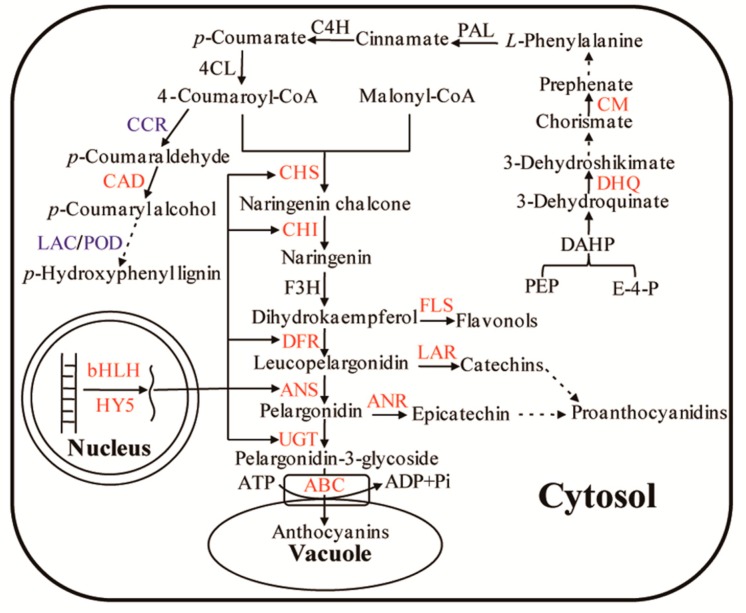
Molecular regulatory network of anthocyanin metabolism in purple leaves of Zijuan tea. Compared with green leaves, fourteen differentially expressed proteins were up-regulated in purple leaves (in red ink) including ABC, UGT, ANS, DFR, LAR, ANR, FLS, CHI, CHS, CM, DHQ, CAD, bHLH and HY5, and two proteins were down-regulated (in blue ink) including CCR and LAC/POD. Solid arrows represent single-step reactions (direct conversions); dashed arrows represent conversions through multiple steps. Abbreviations: ABC: ATP-binding cassette; ADP: adenosine diphosphate; ATP: adenosine triphosphate; UGT: UDP-glucosyl transferase; ANS: anthocyanidin synthase; DFR: dihydroflavonol 4-reductase; LAR: leucoanthocyanidin reductase; ANR: anthocyanidin reductase; FLS: flavonol synthase; F3H: flavanone 3-hydroxylase; CHI: chalcone isomerase; CHS: chalcone synthase; C4H: cinnamate acid 4-hydroxylase; 4CL: 4-coumarate-CoA ligase; CCR: cinnamoyl-CoA reductase 1; CAD: cinnamyl alcohol dehydrogenase; LAC: laccase-6; POD, peroxidase; PAL: phenylalanine ammonialyase; CM: chorismate mutase; DHQ: 3-dehydroquinate dehydratase; PEP: phosphoenolpyruvate; E-4-P: erythritolpyruvate-4-phosphate; DAHP: 3-deoxy-2-arabino-heptulosonate-7-phosphate synthase.

Phenylalanine (Phe) is a primary substrate in anthocyanin synthesis. As such, Phe biosynthesis plays an important role in anthocyanin accumulation in tea plant cells. In the Phe synthetic pathway, dehydroquinic acid dehydrogenase (DHQ) and chorismate mutase (CM) are two key enzymes that catalyze the conversion of phosphoenolpyruvate and erythritol-4-phosphate to Phe [33]. DHQ catalyzes 3-dehydrogenase quinic acid to generate shikimic acid. The conversion of chorismic acid, a major substrate in Phe biosynthesis, to prephenate is catalyzed by CM. Subsequently, the conversion of prephenate to Phe is catalyzed by prephenate dehydratase and transaminase. However, the conversion of chorismic acid to tryptophan is also catalyzed by anthranilate synthase. Thus, the increased expression of CM is necessary to catalyze the conversion of chorismic acid to Phe (Figure 5). In our study, we discovered that the abundance of DHQ and CM was higher in purple leaves compared to green leaves. This finding suggested that the amount of accumulated Phe in purple leaves was higher than that in green leaves, which could provide sufficient substrate for anthocyanin biosynthesis in purple leaves of Zijuan tea.

### 3.4. Regulation of Branched Pathway in Anthocyanin Biosynthesis

The intermediates of anthocyanin biosynthetic pathway can be used to synthesize other secondary substances. For example, 4-coumaric acyl-CoA is the primary precursor of lignin, alkaloids. The conversion of 4-coumaric acyl-CoA to *p*-coumaraldehyde can be catalyzed by CCR, which is the key enzyme regulating the lignin synthesis pathway [34,35]. The inhibition of *CCR* expression significantly reduces the amount of lignin production [36,37]. POD and laccase are two important enzymes in lignin monomer oxidization and polymerization. Sato et al. [38] found that *POD* gene expression is closely related to lignin synthesis. Liang et al. [39] discovered that laccase-6 (*LAC*) participates in lignin synthesis in *A*. *thaliana*. The lignin concentration in *LAC* mutants is significantly reduced, but their soluble proanthocyanidins remarkably accumulate. These results implied that metabolites likely participate in anthocyanin and other flavonoid synthesis pathways when lignin synthesis is suppressed. In our study, CCR1, POD15, and LAC6 had lower abundance in purple leaves (Table 1). Lignin concentration of purple leaves of Zijuan tea was significantly lower than that of green leaves. These results suggested that more 4-coumaric acyl-CoA mainly flowed into anthocyanin biosynthesis in purple leaves, which served as specific regulation mechanism in anthocyanin accumulation of Zijuan tea except for common regulation mechanisms. This regulatory mode was first found in purple foliage tea, though not in the model plant.

## 4. Materials and Methods

### 4.1. Plant Materials

Nine cutting propagation tea trees growing in the tea garden of Fujian Agriculture and Forestry University in Fuzhou, China, were obtained from the mother Zijuan tea tree (*Camellia sinensis* var. *assamica* (Mast.) *kitamura*) in which its genetic identification had been performed and cultivated in the Germplasm Garden of Tea Research Institute, Yunnan Academy of Agricultural Sciences in Menghai, China [29,40]. These nine five-year-old trees were divided into three groups (biological replicates) randomly, each group consisting of three trees. The second and third piece of the purple leaves and the fourth and fifth piece of the green leaves of Zijuan tea trees with similar sizes, colors, and without disease-, insect- and machinery-induced damage, were collected. Total 30 purple leaves and 30 green leaves from each group were harvested and mixed in June 2014, respectively. These leaves were frozen in liquid nitrogen and kept in a refrigerator at −80 °C until use.

### 4.2. Protein Extraction and iTRAQ Labeling

Total protein concentration of the tea leaves was extracted in accordance with a previously described phenol extraction method [41]. Protein powder (10 mg) was solubilized in 250 μL of lysis buffer (7 mol·L^−1^ urea, 2 mol·L^−1^ thiourea, 40 g·L^−1^ 3-[(3-cholamidopropyl)-dimethylammonio] propanesulfonate (CHAPS), and 40 mmol·L^−1^ dithiothreitol (DTT)) in a water bath at 37 °C for 2.5 h. The homogenate was centrifuged at 17,000× *g* for 15 min at room temperature. The supernatant was collected, and the protein concentration was measured in accordance with a previously described procedure [42].

After reductive alkylation was completed, 1 µg·µL^−1^ enzyme liquid was added to 100 µg of protein sample on the basis of trypsin concentration (mass spectrum pure, Promega): protein = 1:20. The sample was incubated at 37 °C for 4 h. This step was repeated twice. After hydrolysis occurred, the resulting peptides were dried through vacuum centrifugation. The peptides were then re-dissolved in 0.5 mol·L^−1^ tetraethyl ammonium bromide. One unit of iTRAQ label was thawed at room temperature and reconstituted in 70 μL of isopropanol. Peptides from green and purple leaves were labeled with iTRAQ tag, respectively. The peptide samples were incubated for 2 h, pooled together, desalted using Sep-Pak Cartridge (Waters Assoc., Milford, MA, USA), and dried through vacuum centrifugation until use.

### 4.3. Strong Cation Exchange (SCX) Fractionation

The peptide samples were separated using a polysulfoethyl SCX separation column (2.1 mm × 100 mm) in a Shimadzu LC-20AB liquid phase system (Shimadzu, Kyoto, Japan). The peptide samples were re-dissolved in buffer A (25% (*v*/*v*) acetonitrile (ACN) containing 10 mmol·L^−1^ KH_2_PO_4_, pH 2.8. The samples were gradiently eluted at a rate of 200 μL·min^−1^ with buffer A for 10 min, with 0–35% buffer B (25% (*v*/*v*) ACN containing 10 mmol·L^−1^ KH_2_PO_4_ and 350 mmol·L^−1^ KCl, pH 2.8) for 30 min, and with 35–80% buffer B for 2 min. Elution was monitored at an absorbance of 214 nm, and 30 fractions were collected. Each fraction was desalted in a Strata X column and dried through vacuum centrifugation.

### 4.4. LC-ESI-MS/MS Analysis

A solution of each fraction was injected into a 20AD HPLC system (Shimadzu, Kyoto, Japan) combined with the liquid phase of the mass spectrometer and equipped with Micromass C18 column (5 µm, 300 Å, 0.1 mm × 15 mm) for LC-MS/MS analysis. Each fraction (2.25 g) was eluted at a rate of 2 μL·min^−1^ with buffer A (2% (*v*/*v*) ACN and 0.1% (*v*/*v*) formic acid) for 15 min. Afterward, each eluted fraction was separated at a rate of 200 nL·min^−1^ with 5% (*v*/*v*) buffer D (98% (*v*/*v*) ACN and 0.1% (*v*/*v*) formic acid) for 1 min, a linear gradient of buffer D from 5% (*v*/*v*) to 35% (*v*/*v*) for 65 min, a linear gradient of buffer D from 35% (*v*/*v*) to 80% (*v*/*v*) for 5 min, and 80% (*v*/*v*) buffer D for 5 min.

The mass spectrum data were collected using TripleTOF 4600 (AB SCIEX, Concord, ON, Canada) with Nanospray III source (AB SCIEX) as an ion source and a radiator of a specific spraying needle (New Objectives, Woburn, MA, USA) composed of a quartz material. The instrument of TripleTOF 4600 was run with peptide mass fingerprint recognition mode enabled, with the following parameter sets: electrospray ionization source, 2.5 kV; nitrous pressure, 30 psi; atmospheric pressure, 15 psi; outlet temperature, 150 °C; scan modes with a reflection and pulse frequency, 11 kHz; inspection frequency, 40 GHz; and normalized collision energy, 35 ± 5 eV; with at least twice the collision frequency of the same parent ion for 18 s.

### 4.5. Bioinformatics Analysis

MS/MS data were analyzed using MASCOT (V2.3.02, Matrix Science, London, UK) and compared with those in National Center for Biotechnology Information non-redundant protein sequences (NCBI-nr) database (release date: 2010 07 01) and Viridiplantae (Green Plants) databases. Data were also searched from the transcriptome database of Zijuan tea by using MASCOT. The engine parameters of MASCOT were set as follows: search type, MS/MS; threshold of ion score cutoff, 0.05 (with 95% confidence); MS/MS fragment mass tolerance, ±0.1 Da; enzyme-peptide tolerance in trypsin, 0.05 Da; monoisotopic mass values; peptide charge states, +2 and +3; and unrestricted protein mass.

The relative intensities of the reported ions were used to determine the abundance of proteins in two groups, namely, green leaves (group G) and purple leaves (group P). Group G was used as a reference in Isobaric Labeling Multiple File Distiller and Identified Protein iTRAQ Statistic Builder. Proteins were considered differentially expressed when they exhibited more than 1.5-fold or less than 0.6-fold change in abundance (*p* < 0.05) in the two groups. Differentially expressed proteins were then subjected to Gene Ontology (GO) and Kyoto Encyclopedia of Genes and Genomes (KEGG) metabolic pathway analyses.

### 4.6. Chlorophyll, Proanthocyanidin, Anthocyanin, and Lignin Analyses

Chlorophyll was extracted from the freeze-dried leaves by using 80% acetone, and the chlorophyll levels were measured in accordance with a previously described method [43]. The absorbances of the extracts were determined at 470, 645 and 663 nm.

Proanthocyanidin concentrations were determined by the method of Skerget et al. [44] with minor modifications. The freeze-dried leaves (0.2 g) were ground in liquid nitrogen in a mortar and ultrasonically homogenized in 5 mL of 95% ethanol at 40 °C for 1 h. The homogenate was centrifuged at 10,000× *g* for 10 min. The supernatants were collected in a 25 mL volumetric flask. Extraction was performed thrice. The sample solutions were diluted with 95% ethanol to obtain a final volume of 25 mL and then mixed. A sample solution (0.5 mL) was mixed with 1.3 mL of 95% ethanol, 6 mL of 5% (*v*/*v*) vanillin–(hydrochloric) acid, and 0.2 mL of 20 g·L^−1^ ammonium iron (III) sulfate solution in 20 mol·L^−1^ HCl and incubated at 95 °C for 1 h. The absorbance of the mixture was determined at 546 nm. The proanthocyanidin concentrations in the sample were calculated on the basis of a previously established standard curve showing the correlation between proanthocyanidin concentrations and absorbances.

Anthocyanin concentrations were measured using the previously stated method described by Zheng et al. [45] with slight modifications. The freeze-dried leaves (0.1 g) were cut and placed in a volumetric flask and soaked twice in 10 mL of 95% ethanol containing 0.1 mol·L^−1^ hydrochloric acid at 60 °C for 30 min. The extracts were further diluted with 0.1 mol·L^−1^ hydrochloric acid and ethanol to obtain a final volume of 25 mL. The absorbance of the extracts was determined at 530, 620 and 650 nm using a solution containing 0.1 mol·L^−1^ hydrochloric acid and ethanol as a blank control, and finally, the concentrations anthocyanin in the leaves were calculated [45].

Lignin concentrations were measured based on a method reported previously with slight modifications [46]. The freeze-dried leaves (0.2 g) were ground into fine powder in liquid nitrogen and ultrasonically homogenized in 8 mL of 80% (*v*/*v*) ethanol at 80 °C for 10 min. The homogenate was centrifuged at 10,000× *g* for 10 min, and the supernatants were discarded. These steps were repeated thrice. The pellet was washed three times with ethanol and *n*-hexane (*v*:*v* = 1:1) and dried at 50 °C until absorbance analysis. The pellet was solubilized in 0.5 mL of 25% (*v*/*v*) acetyl bromide at 70 °C for 45 min. 0.9 mL of 2 mol·L^−1^ NaOH, 5 mL of acetic acid, and 0.1 mL of 7.5 mol·L^−1^ hydroxyamine hydrochloride were sequentially added to the resulting solution. The mixture was homogenized and centrifuged at 10,000× *g* for 10 min. The supernatants were collected; 2.9 mL acetic acid was added to 0.1 mL of the collected supernatant. Absorbance was determined at 280 nm, and the relative lignin concentrations were measured at A_280_·g^−1^ DW.

### 4.7. PAL, CHI and UGT Assays

Fresh leaf samples (1 g) were ground into powder using liquid N_2_ with a mortar and pestle, and then homogenized with 5 mL Na_2_HPO_4_/KH_2_PO_4_ buffer (pH 7.0) including 5 mmol·L^−1^ β-mercaptoethanol, 5% (*w*/*v*) polyvinyl-polypyrrolidine (PVPP), 50 mmol·L^−1^ ascorbic acid and 0.05% (*v*/*v*) Triton X-100. The homogenate was centrifuged at 20,000× *g* for 20 min at 4 °C. The supernatant was collected as the enzyme extract to measure the activities of phenylalanine ammonialyase (PAL), chalcone isomerase (CHI) and UDP-glycosyl transferase (UGT) in accordance with the protocol described by Huang et al. [47]. The activity of PAL was determined by examining the production of trans-cinnamic acid from deamination of phenylalanine. One unit (U) of PAL activity was defined as an increase of 0.01 in absorbance at 290 nm per minute. Meanwhile, the activity of CHI was measured using the degradation of chalcone at 381 nm, 34 °C. One unit (U) of CHI activity was defined as a decrease of 0.01 in absorbance at 381 nm per hour. In addition, the activity of UGT was analyzed using the reaction of quercetin and UDP-galactose at 351 nm, 30 °C. One unit (U) of UGT activity was defined as a change of 0.001 in absorbance at 351 nm per hour.

### 4.8. Western Blot Analysis of CHS

Western blot was conducted to assess the different expression levels of CHS protein between green and purple leaves. Approximately 90 μg of protein samples was loaded onto 12% SDS-PAGE, processed through electrophoreses for 3 h, and transferred to polyvinylidene fluoride (PVDF) membranes (Millipore, Billerica, MA, USA) in accordance with a previously described method [48]. Ponceau-S staining was used as loading control. After transformation was completed, the membranes were blocked with fat-free milk, incubated with primary antibody (rabbit polyclonal anti-CHS) at room temperature, and washed with TBST buffer (10 mmol·L^−1^ Tris-buffered saline (TBS), 0.1% (*v*/*v*) Tween-20, pH 7.6). Afterward, the membranes were incubated with secondary antibody (horseradish peroxidase (HRP)-conjugated goat anti-rabbit IgG) and immunoreactive bands developed with 3,3′-diaminobenzidine and quantified using software Image J (java image processing program NIH image, Bethesda, MD, USA) and three independent experiments were performed.

### 4.9. RNA Isolation and qRT-PCR Analysis

A modified cetyl trimethyl ammonium bromide (CTAB) method was employed to isolate RNA from the green and purple leaves of Zijuan tea [49]. After that, cDNAs were synthesized for qRT-PCR using the Reverse Transcriptase kit reagents (TaKaRa, Tokyo, Japan) according to manufacturer’s protocol. qRT-PCR was performed in a CFX Connect™ (Bio-Rad Laboratories, Inc., Hercules, CA, USA) platform. The primers were designed on the basis of previously obtained transcriptome data via Primer Premier 5.0 (PREMIER Biosoft International, Palo Alto, CA, USA) (Table 2). Each quantitative reaction was carried out in a 10-μL reaction mixture with 5.0 μL of 2× SYBR Premix Ex Taq™ II (TaKaRa), 1.0 μL of diluted cDNA template, 0.2 μL of each primer (10 μmol·L^−1^), and 3.6 μL of DNase-free water. Amplification was conducted using two-temperature cycling protocols with 30 s at 95 °C followed by 40 cycles of 5 s at 95 °C and 34 s at 60 °C. After the completion of each amplification, melting curve analysis was performed with temperature ramping from 65 to 95 °C (rising by 0.5 °C per step with a 5 s rest) to confirm the specificity of the PCR. Three independent replicates of the samples were prepared. The relative expression level of each gene of the corresponding protein was normalized to *Actin* gene. A relative quantitative computing method (2^−ΔΔ*C*t^ method) was used to quantify gene expression [50].

### 4.10. Statistical Analyses

Statistical analyses were performed using ANOVA to determine significant differences among group means. Data from three independent replicates of each treatment were presented as means ± standard deviation (SD). A *p* value less than 0.05 was considered statistically significant.

## 5. Conclusions

The regulations of anthocyanin biosynthesis were studied based on changes in protein abundances between the purple and green leaves of Zijuan tea using iTRAQ and physiological and biochemical analyses (Figure 5). The transcription levels of anthocyanin synthesis-related genes, including *PAL*, *CHS*, *CHI*, *DFR* and *ANS* in purple leaves, were high due to activation of the transcription factors bHLH and HY5. This led to the high abundance or activity levels of their relative proteins (enzymes) which resulted in enhanced anthocyanin synthesis and accumulation in the cell vacuoles of the purple leaves. Furthermore, anthocyanin monomers can also be modified through glycosylation to form anthocyanins that accumulate in cell vacuoles through the regulation of UGT and ABC transporters in cells. A specific regulatory mechanism in anthocyanin accumulation was found in the leaves of Zijuan tea. The higher abundance levels of DHQ and CM implied that sufficient phenylalanine substrate was supplied for anthocyanin synthesis. Lignin synthesis was also reduced while CCR, POD and LAC abundances were decreased, which indicated that numerous intermediate metabolites flow to the anthocyanin synthesis pathway. This study proposed a potential regulatory network of anthocyanin metabolism in Zijuan tea.

## Figures and Tables

**Table 1:** Differential proteins involved in anthocyanin metabolism in purple leaves of Zijuan tea.

No.	Protein Name ^a^	Sequence Name ^b^	Accession No. ^c^	*E*-Value ^d^	Similarity	G ^e^	P ^f^	Fold
**Anthocyanin metabolism**								
1	chalcone synthase	c38979.graph_c0	gi|320117902	0	94.45%	1	3	3
2	chalcone isomerase	c22272.graph_c0	gi|297736495	5.23 × 10^−121^	91.30%	1	2.7	2.7
3	chalcone isomerase	c38282.graph_c0	gi|325551315	9.70 × 10^−149^	90.75%	1	2.3	2.3
4	dihydroflavonol 4-reductase	c30221.graph_c0	gi|6009511	0	93.25%	1	1.5	1.5
5	anthocyanidin synthase	c13490.graph_c0	gi|378749118	0	92.50%	1	1.8	1.8
6	UDP-glucosyl transferase 88A1	c41584.graph_c0	gi|508711676	5.32 × 10^−153^	71.00%	1	2.6	2.6
7	abc transporter b family member 8	c30601.graph_c0	gi|359484339	0	90.00%	1	1.8	1.8
8	flavonol synthase/flavanone 3-hydroxylase-like	c30094.graph_c0	gi|76786311	0	88.30%	1	2.4	2.4
9	leucoanthocyanidin reductase	c29035.graph_c0	gi|326380568	0	86.55%	1	3.4	3.4
10	anthocyanidin reductase	c36734.graph_c0	gi|294847480	0	93.10%	1	2.4	2.4
**Transcription factor**								
11	transcription factor bhlh135	c13939.graph_c0	gi|508711524	3.92 × 10^−47^	92.50%	1	1.7	1.7
12	transcription factor bhlh66-like	c38737.graph_c0	gi|508782323	1.30 × 10^−78^	67.20%	1	2.2	2.2
13	transcription factor hy5	c31532.graph_c0	gi|470110394	6.94 × 10^−57^	87.45%	1	2.5	2.5
**Shikimic acid pathway**								
14	bifunctional 3-dehydroquinate dehydratase shikimate chloroplastic-like	c27480.graph_c0	gi|259479224	0	87.55%	1	1.5	1.5
15	bifunctional 3-dehydroquinate dehydratase shikimate chloroplastic-like	c29706.graph_c0	gi|225451146	0	84.15%	1	2.2	2.2
16	chorismate mutase chloroplastic	c24623.graph_c0	gi|460372757	1.24 × 10^−44^	70.80%	1	1.8	1.8
**Lignin synthesis**								
17	Predicted: Cinnamoyl-CoA reductase 1	c41372.graph_c0	gi|225452438	0	91.05%	1	0.6	0.6
18	cinnamyl alcohol dehydrogenase	c34762.graph_c0	gi|332384181	0	91.80%	1	1.6	1.6
19	Laccase-6 (Precursor)	c35053.graph_c0	gi|297737720	0	85.90%	1	0.4	0.4
20	peroxidase 15-like	c41476.graph_c1	gi|462402484	4.81 × 10^−147^	76.85%	1	0.4	0.4

^a^ Proteins identified by isobaric tag for relative and absolute quantification (iTRAQ); ^b^ Sequence number of the identified proteins in transcriptome data acquired earlier in our lab; ^c^ Accession number of the identified proteins in National Center for Biotechnology Information non-redundant protein sequences (NCBI-nr) database; ^d^ A parameter to evaluate the probability that other peptides matched the protein; ^e^ Relative expression level of proteins in green leaves of Zijuan tea; ^f^ Relative expression level of proteins in purple leaves of Zijuan tea. abc: ATP-binding cassette.

**Table 2:** The primers used for quantitative real-time PCR (qRT-PCR) analysis.

Genes	Primer Sequence(5′→3′)
*Actin*	GGCAGATAGATGCTTATGTAGGTG
	TGTTTGCTTTAGGGTTGAGTGG
*CHS*	AGTGGAGGAAGTGAGGAGGG
	CGCTGTTAGTAATGCGGAAGT
*CHI*	TCCAAGCCCTTCTTCCTCG
	GACCCGTGAATGGCAAAATC
*DFR*	AGAGCAGGGAGGCTTGTATG
	GAGTATTTGGACCGATGTGG
*FLS*	ATACAGGGGAGTGACAGAGGAAT
	CATTGGGGACAAGTAAAGTGAGA
*ANS*	ACGAGGGCAAATGGGTCA
	TCCTTGGGTGGTTCGCAGA
*UGT*	AATCTGTGGTGTCCGTTTGCTTC
	TCTTCGCTGTCTTCTTTGTCTACTT
*LAR*	TGCGGCGATTGATAGAAG
	GCAGGATGGTCGGAAATG
*bHLH*	CCCGAGATTCGCAATAGGC
	TGTCGCTGAGATCATCCACTTC
*POD*	CGGGCTGGATGCTTGACTGT
	CCCTGCTTGTTCTGGAGGTTAG
*CCR*	AGCAATGGTTGTCGGTCCTC
	ACCTGGTAAGTCGGCGTAAAT
*CAD*	CCTCGGTCCAGACGATTACG
	CAACAAAGAAAGGTATGGCTCAAGT
*DHQ*	GAAGCCTGAAAAGGTCAAAATC
	TTGGCACAAAGTATGCGAGA
*PAL*	ACACTTTATGTGCCCAAGACCC
	GCTTCCGATACTCCGCTACCA
*ABC*	GGTTATGCTGAGCCTGGTAGT
	CCGTTGAGGAGAATAGTGCC
*HY5*	AGGGTCGGTGTCTTTCAG
	GTATGCCTTCTTCCTTTCC
